# One stop crisis centres: A policy analysis of the Malaysian response to intimate partner violence

**DOI:** 10.1186/1478-4505-9-25

**Published:** 2011-06-21

**Authors:** Manuela Colombini, Siti Hawa Ali, Charlotte Watts, Susannah H Mayhew

**Affiliations:** 1 Department of Population Studies, London School of Hygiene and Tropical Medicine, UK; 2 School of Health Sciences, Universiti Sains Malaysia, Malaysia; 3 Department of Global Health and Development, London School of Hygiene and Tropical Medicine, UK

## Abstract

**Background:**

This article aims to investigate the processes, actors and other influencing factors behind the development and the national scale-up of the One Stop Crisis Centre (OSCC) policy and the subsequent health model for violence-response.

**Methods:**

Methods used included policy analysis of legal, policy and regulatory framework documents, and in-depth interviews with key informants from governmental and non-governmental organisations in two States of Malaysia.

**Results:**

The findings show that women's NGOs and health professionals were instrumental in the formulation and scaling-up of the OSCC policy. However, the subsequent breakdown of the NGO-health coalition negatively impacted on the long-term implementation of the policy, which lacked financial resources and clear policy guidance from the Ministry of Health.

**Conclusion:**

The findings confirm that a clearly-defined partnership between NGOs and health staff can be very powerful for influencing the legal and policy environment in which health care services for intimate partner violence are developed. It is critical to gain high level support from the Ministry of Health in order to institutionalise the violence-response across the entire health care system. Without clear operational details and resources policy implementation cannot be fully ensured and taken to scale.

## Background

Globally, evidence on interventions seeking to address violence against women (VAW) by integrating specific services at the health sector level remains fairly limited. There are few well evaluated examples of violence-service initiatives in Southeast Asia, where many focus on rape and are run by NGOs [1]. The Malaysia One Stop Crisis Centre (OSCC) model is relatively unique in its implementation of a large scale model for violence-response, and other countries in the South East Asian region are replicating this approach [2-4]. However, little data are available on the processes and influences that led to its creation and expansion to national level.

Malaysia was among the first countries in the region to adopt a law on partner violence: the Domestic Violence (DV) Act of 1994 [5]. National prevalence data on violence against women (VAW) is still not available today, though the University Sains Malaysia is currently undertaking a national prevalence study on VAW. However, anecdotal evidence suggests that intimate partner violence (IPV) and non-partner sexual violence are quite widespread across the country [6]. More than 2000 abuse cases are reported each year to the Police Department and other agencies [7] (table 1); though, they represent only the tip of the iceberg.

**Table 1:** Statistics for rape, domestic violence and child abuse cases of 3 organizations: OSCC, Police and Welfare Departments from years 2004 - 2006

Organisation/Year	2004			2005			2006		
	
	Rape	IPV	CA*	Rape	IPV	CA	Rape	IPV	CA
***OSCC***	na	na	na	1,099	1,851	160	1,454	2,159	214

***Police Department***	1,765	3,101	148	1,931	3,093	189	1,561	2,171	108

***Welfare Department***	na	560	1,656	na	421	1,800	na	463	1999

Source: Ministry of Health, Ministry of Women and Community Development and Department of Social Welfare, Malaysia.

*CA: Child Abuse

Recent studies suggest that IPV is also quite prevalent in neighbouring countries like Thailand, Philippines and Indonesia [8-10].

Although rape crisis services and a related protocol were available since late 1986 [11], a more comprehensive health sector response to violence against women started in 1994, with the first One-Stop Crisis Centre (OSCC) established in Kuala Lumpur; and in 1996, with a specific directive of the Ministry of Health [12]. This paper employs a policy analysis of the Malaysian OSCC model in order to illumine the driving forces behind its creation and scale-up and provide lessons not only to improve the health care to abused women in Malaysia, but also to inform and influence the development of a health sector response to partner violence in other countries that are struggling with creating and scaling-up similar models of care.

## Methods

The paper explores the processes, actors and other influencing factors behind the development and the national scale-up of the OSCC directive in Malaysia. It first describes the contextual factors and processes that led to the enactment of the DV Law and the establishment of a pilot OSCC service. Then it focuses on the roll out of the policy at national and district levels and what factors and actors influenced it. It concludes by discussing whether establishment of OSCCs is a suitable way of addressing VAW, especially at A&E.

This study is the result of a policy analysis conducted in Malaysia in 2007 which involved analysis of legal, policy and regulatory framework documents, and in-depth interviews with key informants from governmental and non-governmental organisations in two States of Malaysia, namely Kelantan and Penang. A total of 20 interviews were conducted between January and April 2007, details shown in Table 2.

**Table 2:** Respondents interviewed

State/level	Type of Respondent	Number of respondents
**Penang State**	Policy makers from the Department of Health	3
	Representatives from collaborating agencies (police and social welfare)	2
	NGO representatives	2
**Kelantan State**	Policy-makers from the State Health Department	2
	Representatives from collaborating agencies (police and social welfare)	2
	Representative from local NGO	1
**National Level**	Senior-policy makers at Ministry of Health	3
	NGOs representatives	4
	Health provider from the capital's hospital	1

In-depth interviews were conducted in English, tape-recorded, transcribed and uploaded to NVIVO (N7), a qualitative software package, for coding and managing narrative text for analysis.

The use of a policy analysis lens permits a detailed analysis of actors and processes that help to understand the course Malaysia took in establishing OSCCs and the challenges it has faced in scaling them up. The policy analysis model used in this article - or health policy triangle model - consists of four elements that are essential to analyse factors influencing the policy process and the implementation of health policies: 1) actors (individual, groups or organizations), 2) content, 3) context (situational, structural, cultural and international factors), and 4) processes (problem identification, policy formulation, policy implementation and evaluation) [13,14].

The four components from the policy analysis framework were employed closely to examine existing policies and guidance documents on IPV in Malaysia, and processes used by various actors to develop and scale-up the OSCC services. The focus on these elements helped analyse the various approaches used by the health sector to implement IPV services. In particular, it helped answer questions like: "who was supportive of which intervention? Who was against it and why? How did the organization manage to win support from opposing agencies? What did the policy state?

Beside the "policy triangle" approach, in our discussion we also draw on Hall and Kingdon's models of agenda setting [15,16] and the implementation frameworks, principally the *top-down *(and more prescriptive) [17] and *bottom-up *approaches, which are more focused on the role that different individuals play in policy implementation particularly lower-level officials or "street-level bureaucrats" [18]. We use these to help describe and explore how violence against women became a legitimate policy issue for the Malaysian government, how the OSCC became a national policy, and how it was scaled-up at State and district level.

### Document analysis

A document analysis of relevant Malaysian laws, policies, regulations, and policy statements on domestic violence and related issues was undertaken to analyse content, status of implementation and policy implications on the provision of services. The review of policy documents and existing data and reports helped inform the national level analysis of actors and structural factors and was used as part of the background information to inform the analysis of the interviews with key informants and policy-makers.

In-depth interviews were conducted with key informants, including policy makers, women's influential NGOs, and representatives from government agencies working on IPV (at national and regional level), to identify the policy and organisational context, training, follow up, supervision, quality assessments and other measures that are taken to develop and sustain the integration of services for abused women. Interviews also helped understand where the emphasis on domestic violence lays at national level (e.g. strong focus on legal issues or on public health ones) and how this may impact on the delivery of services.

Semi-structured topic guides for the policy-makers included the following questions:

- Government and Ministry of Health (MOH) stance towards IPV: to understand whether there is a national support or not, and whether IPV is considered a priority issue

- history of policy process (asked primarily at national level): to understand the processes and actors behind the OSCC policy implementation

- guidelines and protocol: to get a sense of the guidance on IPV provided by the top management

- training and resources: to get a sense of the emphasis and importance given to training by MOH

- networks and alliances around violence: to understand collaboration process

- perceptions and views around IPV: to get a sense of their views towards IPV across units and agencies

This structure tried to encourage respondents to discuss matters they might have otherwise not revealed without prompting such as their beliefs towards IPV. It also ensured that issues relevant to the research were not completely overlooked and allowed comparison across sites and between States.

### Sampling and recruitment

Snowball sampling was used to identify key informants who were knowledgeable about the research topic and the researched model, with the assistance of the local partners. Respondents were selected according to their profession and their experience with violence issues and their connection with OSCCs. A total of 20 people were interviewed, until we reached theoretical saturation and no more new contacts were suggested by key informants.

Ethical approval has been granted by the Ethical Committees of the LSHTM School and the World Health Organisation. Ethical permission for the study has also been granted by the national ethical review committee in Malaysia. Additional permissions to conduct interviews with health providers in the selected hospitals were also obtained by the State Health Departments in Penang and Kelantan.

Written informed consent was obtained from each participant in the study. Each informant was asked to sign an informed consent form and anonymity of all information was assured by asking them whether they agreed to be quoted in disseminating reports.

An analysis method framework was used to analyse the interview findings, which consists of a content analysis method allowing for a systematic classification and organisation of data by major themes, categories and concepts within a thematic framework [19]. This approach encourages the preservation and integrity of the voices and accounts of the interviewees, keeping the researcher grounded in the data, as the information is summarised and classified within a thematic matrix all along the analysis [19].

## Results

### 1. Front-line actors establish legitimacy of violence as a national problem, shape service protocols and pilot OSCC services

In Malaysia, violence against women (VAW), and intimate partner violence (IPV) in particular, has always been perceived as a private matter. However, during the early 1980s, things began to change due to the rise of several women's organisations and NGOs [12], which was crucial in making violence against women a more public issue [12]. Women's groups addressed issues of violence against women initially through legislative efforts [20]. Analysis of policy and organisational documents showed that, in 1985, a Joint Action Group (JAG) was created as an umbrella group of individual women and five women's NGOs (Women's Aid Organisation, Association of Women's Lawyers, Malaysian Trade Unions Congress Women's Section, University Women's Association and the Selangor and Federal Territory Consumers' Association) working together on violence issues [21]. A timeline of main events described here - resulting from the document analysis - is outlined in Table 3.

**Table 3:** Timeline of main events in the development of DV policy and the establishment of OSCCs

1985	Creation of Joint Action Group against violence against women (JAG) to lobby for a law against domestic violence
1991	JAG presented a Bill on DV to Government
1993	National seminar on "Interagency Management of battered women" held in Kuala Lumpur
1994	Adoption of the DV Bill
1994	First Pilot OSCC in the A&E of the Hospital Kuala Lumpur
1996	DV Bill enacted as a criminal legislation
1996	OSCC protocol was fully adopted at HKL
	Creation of a second OSCC in Penang
	Ministry of Health (MOH) circular on the creation of OSCC at all public hospitals
Late 90's	NGOs in Kuala Lumpur withdrew from OSCC in Kuala Lumpur
2004	MOH official letter to all hospitals to collect information about OSCC

DV: domestic violence; HKL: Hospital Kuala Lumpur

Results from the document analysis showed that in 1989, JAG started lobbying for legal provisions to address cases of domestic violence, as no previous law existed on protecting women from marital abuse [12,22]. It was only in 1994 that the Domestic Violence (DV) bill was tabled in Parliament and finally enacted in 1996 as a criminal legislation [20]. Women's NGOs were catalysts in the adoption of the DV Act and in pushing the issue of intimate partner violence forward in both the public arena and the Government agenda.

In parallel to the development of a legal basis for recognising and responding to DV, front-line health workers and NGO groups were active in trying to establish formal service protocols for managing victims of DV and pushing for a coordinated multi-sector response that led to the establishment of a pilot OSCC. Prior to 1993, services for abused women existed but were provided in an ad hoc manner and in small scale [23,24]. The development of an interdisciplinary model of health care to abused women was heavily influenced by one influential doctor at the General Hospital in Kuala Lumpur (HKL) [Information given during discussions with key informants in Kelantan and Kuala Lumpur]. An interviewee said that the doctor recognised the need to do more than treat physical problems:

[..] she had actually said that when many women who are abused come to the hospital, she saw that they wanted to talk to her more ...[...] the need is more of psychological, counselling rather than fixing a broken arm [...] the doctor said that she had no time to talk to [her], she can only talk about fixing her broken arms and in the hospital they see 300 patients at a time and so she cannot be sitting there doing counselling with the women ...[...] [KL/35, female]

The female doctor pushed for collaboration beyond the health sector with women's NGOs, legal and religious groups [25] (Interviews with key informants, Kuala Lumpur, February-March 2007). In December 1993, a national seminar on "Interagency Management of battered women" was held [25,26]. Subsequently, in 1994, the first OSCC was created as a pilot project at the Kuala Lumpur Hospital to support survivors of physical and sexual violence [27].

Based on the OSCC concept, all services - medical, counselling and police services - would be administered at the hospital itself, within the Accident & Emergency (A&E) Department. Legal aid and religious support would be offered upon referral [25]. The link between clinical services and NGO support was crucial in the establishment of OSCC. As part of the process, some women's advocate volunteers would be on 24-hour call to respond to calls from the A&E to assist abused women and to counsel them when arriving at the hospital [25]. The initial expectation from the medical sector was that they would focus primarily on treatment, while the counselling and long-term support was given by volunteers from women's NGOs. On the other hand, women's NGOs thought that they would only cover the counselling temporarily until more social workers would be seconded to the OSCCs.

*At that time I think we *[NGOs] *were asking for full time workers, you know, full time social workers to be stationed at the One Stop Crisis Centre. As we were providing services, we realize that we shouldn't be doing this on the long term because it should be really the responsibility of the State to provide the social workers to be there. [KL/35, female]*

Though crucial, the nature of the NGO/health partnership - and the subsequent responsibilities - was never really spelled out properly or discussed formally between the two parties. Interviews with key informants made it clear that from the very beginning there seemed to be a misunderstanding between women's organisations and the medical sector on the role to be played by NGOs^1^

*We *[NGOs] *thought that we should slowly back up and it should be now the State player that should be running it effectively without us in there. [...] We told them [the hospital people in charge of OSCC] 'call on us for the running of the gender sensitizing programs, include us in the awareness, but in term of the services then it's your baby, you take care of it'. [KL/36, female]*

On the other hand, the MOH did not seem to absorb the idea that OSCC should be primarily a health package fully supported by public services. This tension became an important factor in the weak scale-up of the initiative.

Between 1994 and 1996, an OSCC protocol was fully adopted at HKL [25], together with hospital guidelines for the management of OSCC cases. The hospital Kuala Lumpur also called for regular interagency meetings with partners from the Police, Social Welfare, Legal Aid and NGOs, which helped improve OSCC services, and, indirectly, forced the government agencies to monitor their services.

Front-line health workers and NGOs/women's groups were instrumental in bringing the issue of violence to the attention of policy-makers, pushing for legal response and showing that OSCCs could work. However, there was a critical breakdown in understanding of the roles and responsibilities between NGOs and Government agencies (hospitals in particular) that was to prove very damaging for sustaining the scaling up of the OSCC model.

### 1. Policy Development: the lack of policy clarity over responsibilities and need for careful balancing of Government - NGO relations

Despite the adoption of the DV Act and the running of a successful pilot of a coordinated model of violence-response at the hospital level, the Ministry of Health had not yet developed a formal policy on the health sector response to violence against women^2^. In 1996, after the development of the first piloted OSCC in Kuala Lumpur, women's NGOs lobbied for the creation of a similar set up in the main regional hospital in Penang. It was only after the creation of this second OSCC (thanks to the NGOs' lobby) that the Ministry of Health became officially involved in activities related to OSCCs and VAW and announced the intention of replicating OSCC structures throughout Malaysia (interview, women's NGO representative, Penang, February 2007).

Subsequent to this event, all the elements were in place for the national legitimisation of OSCC: a well-developed hospital structure, a management protocol, two functioning OSCCs, and the lobbying from the women's NGOs. In the same year (1996), the Medical Development Division of MOH sent out a circular containing clear instructions on how to establish comprehensive services for rape and abuse victims through OSCCs [28] stating that all government hospitals should develop a One Stop Crisis Centre in order to fight violence against women.

Despite being a policy landmark for OSCC creation and the provision of services to abused women, the 1996 MOH circular did not specify how the centres should be created, except that a variety of hospital units should be involved and it should be managed by various agencies in a way that encouraged multidisciplinary coordination [28]. It was unclear who should direct OSCCs, and how to implement them at district facilities with no specialised care and very few NGOs outside the capital city.

*KL *[Kuala Lumpur] *is quite lucky because they have few active NGOs. In many towns they don't, so it's very scattered in terms of accessibility and support*. [PP/22, male]

In reality, it was very much left at the discretion of each hospital's director to develop its own procedures. No further guidelines or procedures have been developed by the Ministry of Health.

Interviewees suggested that the strong women's lobby was instrumental in pushing MOH to support the creation of OSCCs throughout Malaysia.

*[..] I think the women's NGOs were instrumental to push for the OSCC in this country [...] we were very concerned about the kind of treatment women should get if they go for medical help. So I think we were the one who pushed for the setting up of the OSCC*. [PP/10, female]

Furthermore, the partnership that developed between NGOs and hospital professionals and thus with the MOH was crucial for women's NGOs in order to push for integrated services to abused women within the entire health care system. Within the Malaysian hierarchical system, it would have been otherwise impossible for NGOs to penetrate and influence the health sector and the top Ministerial level without the internal support of the hospital (Head of A&E) and MOH. The fact that the initial implementation process was government-led (by MOH and hospital particularly) added credibility to the request for specific services for abused women and to the entire process, and made it acceptable for other government agencies to collaborate (rather than just having NGOs, because of the historical antagonism between women's NGOs and Ministries).

*It *[Interagency Committee] *was coordinated by the hospital people. [...] It was easy because it was a government department, the hospital itself, calling the other government departments [...] because if we [NGOs] were coordinating it and we were calling they [government agencies] would then say 'oh, what do now the NGOs want from us?'. But because it was all State players so the coordination was definitely much easier. [KL/35, female]*

The pilot project in HKL gave legitimacy to the expansion of OSCC services and helped the government recognise violence against women as a national health problem which needed action. However, the lack of policy clarity over roles and responsibilities across the various actors - NGOs, health staff, other agencies - called for a more careful balancing of Government - NGO relations.

### 1. Policy implementation: challenges to scaling-up revealed

The establishment of the OSCC was planned in stages. By the end of December 1996, all State hospitals were to have an OSCC, while all district hospitals should have had one by 1997 [28]. In reality, based upon interviews with key informants and providers, it took much longer for district hospitals to integrate OSCC services and they faced two important challenges.

First, the implementation of OSCCs relied heavily on NGOs providing counselling and support services, but NGOs working on violence issues were in short supply and did not have a presence in many States. More critically, in the late 1990s, NGOs gradually stopped supporting OSCCs actively because their on-site advocacy services could not be sustained in the long term, as they did not receive any additional funding.

Second, as supported by the findings from the document analysis, a crucial flaw in the policy formulation and its rolling out nationally was related to the fact that in the MOH circular, it was clearly stated that no additional budget would be given to hospitals to establish such centres [28]. Key informants also suggested that the implementation of the OSCCs was not a top priority for the MOH.

The OSCCs are physically there but then they are not staffed, they don't have enough human resources. [...] I felt that they [MOH] were not willing to put in extra money. They did not want to fund it.. they didn't see the need of putting in money. I think it is just a political will, it was not their priority, otherwise they would have pumped some money into it. [KL/35, female]

In October 2004, MOH sent a letter to all hospitals to ensure that they had set up OSCCs properly and to collect information about these services [29]. The emphasis was on the physical infrastructure and layout of the room, rather than on managerial issues and processes around the OSCC. To date, this has been the last ministerial directive sent by MOH on OSCCs.

Our key informant interviews suggested that without the full support from NGOs on site and from the top policy level, the model got "*more medical with less focus on women's needs" (*Interview with key informant, Kuala Lumpur, April 2007) and its survival became more and more dependent on the existence of "*in-house champions*" who would coordinate and support the programme within the hospital. Without such medical figure on-site, without additional resources from the policy level and without the support of any women's groups, some OSCCs were left with a fragile support framework or would "*disappear silently*" (email exchange with key informant from Kelantan, August 2008).

The lack of financial commitment on the part of the Government was a major flaw. Nonetheless, the dedication of front-line staff was such that most hospital did in fact establish OSCCs, but funding constraints meant they were quite variable in their ability to roll out services. Consequently, there was increasing "medicalisation" of services and disconnection of broader NGO inputs, leaving many OSCCs with minimal support in place.

## Discussion

The study findings confirm other studies' evidence about the importance of looking at the interaction of actors, context and content [14] as a starting point for investigation. In particular, the research provided a key lesson for each stage of the policy cycle in relation to the Malaysian response to domestic violence. While this framework is useful, a range of other policy theories also give insight for understanding what was going on in the establishment and expansion of OSCCs in Malaysia [15,16,18,30,31].

First, the study confirms that the 1994 DV Law gave IPV what policy theorists in the literature refer to as "*legitimacy*" [15]. With a legitimate problem defined, the success of the OSCC pilot meant that OSCCs acquired national credibility as a feasible policy solution to the VAW problem, resulting in official MOH support on the issue. Similar to Hall [15], John Kingdon's theory of agenda setting [16] holds that in addition to a legitimate problem and a defined solution (Hall's 'feasibility'), political will (or what Hall calls 'support') is needed for policy change. The joint push by NGOs and influential hospital staff on the importance of OSCCs showed how front-line staff - the so-called Lypsky's "*street level bureaucrats*" [18], influenced government thinking, which resulted in the MOH drawing up a formal policy for national scale-up and disseminating this through the 1996 Directive. Despite being a watershed, the 1996 policy was inadequate for replicating OSCCs - for reasons that implementation theories, including Lipsky's, help to explain.

Second, the creation and scale-up of OSCCs was arguably a mix between bottom-up and top-down approaches, where initial agenda setting came from bottom-up coalitions of NGO and health staff, while detailed policy formulation was left to MOH. The initial joint partnership between hospital front-line staff and women's NGOs was pivotal in bringing IPV onto the policy agenda. On the other hand, although the formulation of the 1996 policy itself was top-down and elaborated by the MOH, it failed to provide the operational details or resources to ensure policy implementation. The sole policy document of 1996 had no clear objectives, no proper guidance on replication, no funds, and no monitoring system. Like many other studies and theories, this case confirms the shortcomings of a top-down approach that fails to engage the 'street level bureaucrats' [30,32]. Moreover, the lack of allocated budget devoted to the creation and expansion of the OSCCs was equally to blame for the failure of the implementation of the MOH policy.

Third, the NGO-health provider coalition was also not successful in sustaining its influence through policy formulation. During the establishment of the first OSCCs the roles of NGOs had not been clearly defined and the hospitals expected NGOs to take on more than they could afford to and with no financial support from the State. Once NGOs had been drawn into service implementation their public advocacy role declined which affected public support. Moreover, a rift was opening up between NGO and hospital staff over who should be responsible for delivering and funding the non-clinical services of the OSCCs, which meant NGOs lost their influence at an advocacy or policy level. At the same time, political concerns had moved away from DV and by the late 1990s rolling out OSCCs was no longer a priority for the MOH, losing what Hall calls "support" or political will [15].

Another critical issue debated in the literature is whether OSCCs are the best model to respond to IPV [33], even if assuming the challenges identified in the paper are dealt with. Little is published on OSCCs effectiveness or other similar complex models of health care response to IPV [9,10,33]. Existing evidence shows that other entry points - such as primary health care, antenatal and family planning services - can be successful in reaching women who experienced violence [34-36]. Most of the trial evidence in the North for system-based interventions to improve health care responses to IPV were based in primary care. Some of these may be more relevant to low income countries than the acute sector-based OSCC model.

However, it would be a pity to dismantle OSCC services in Malaysia because of the paucity of evaluated data. OSCCs should instead be developed and strengthened, especially at tertiary and secondary hospital level. Efforts should also be putting in establishing new services at primary health care. In Malaysia, if properly resourced, OSCCs can provide effective care to abused women, especially in rural areas where there are no other services. Nonetheless, this model may not be feasible in low income countries where the health infrastructure, the human and financial resources are not available to run such specialized and integrated response, and primary care responses may be more suitable.

## Conclusions

There are a number of lessons from this case-study. First, the Malaysia case shows how powerful a partnership between NGOs and health staff can be for influencing the legal and policy environment in which OSCCs are developed. NGOs knew how to lobby government and influence public opinion while senior hospital staff were more influential with the MOH and provided professional evidence of the need for specialist services. Separately it is less likely they could have influenced both the DV Act and the establishment of the OSCC pilot. Second, it is of critical importance to gain high level support from the Ministry of Health in order to institutionalise the response across the entire health care system. Without MOH buy-in and allocated resources a national scale-up policy would not have been developed. However, the challenge is to sustain this support through to full implementation and secure government commitment to properly resourcing the OSCCs throughout the country. Reactivating the partnership between NGOs and hospital staff could be critical in achieving this.

Another critical issue to consider is whether the integration of OSCCs within A&E was a suitable way to deliver services to abused women in a middle income country. Given the Malaysian context, A&E represents the best first entry point for many women as it is open 24 hours, no prior appointment is needed and there is at least a semi-functioning OSCC network throughout the country with many frontline staff still committed to providing services. However, primary health care responses to GBV should also be strengthened to create synergies between the two approaches.

## Competing interests

The authors declare that they have no competing interests.

## Authors' contributions

MC conceived the study, carried out the policy analysis, conducted the interviews with policy-makers and drafted the manuscript. SM participated in the development, design and coordination of the study, supported the conduction of the policy analysis and helped draft the manuscript. SHA participated in the policy analysis and supervised the fieldwork. CW conceived the study, and participated in its design and coordination and helped draft the manuscript. All authors read and approved the final manuscript.

## Endnotes

^1. ^From interview with key informant from NGO in Kuala Lumpur, April 2007 and email exchange with key informant in Kelantan in August 2008.

^2. ^This was despite having had operational procedures on management of rape and other sexual violence abuse since the late 1980s (Ministry of Health, Malaysia, 1988).
